# Identification of QTL hot spots for malting quality in two elite breeding lines with distinct tolerance to abiotic stress

**DOI:** 10.1186/s12870-018-1323-4

**Published:** 2018-06-04

**Authors:** Andriy Kochevenko, Yong Jiang, Christiane Seiler, Korana Surdonja, Sonja Kollers, Jochen Christoph Reif, Viktor Korzun, Andreas Graner

**Affiliations:** 1 Leibniz-Institute of Plant Genetics and Crop Plant Research (IPK), Corrensstr. 3, OT Gatersleben, D-06466 Seeland, Germany; 2 KWS LOCHOW GmbH, Ferdinand-von-Lochow-Str. 5, 29303 Bergen, Germany

**Keywords:** QTL mapping, Malting quality, Drought-tolerance, Spring barley, Elite breeding lines, iSelect array

## Abstract

**Background:**

Barley (*Hordeum vulgare*) is an important crop cultivated across the world. Drought is a major abiotic factor compromising barley yield worldwide, therefore in modern spring barley cultivars superior seed and malting quality characteristics should be combined with reasonable level of drought tolerance. Previously we have identified a number of barley lines demonstrating the superior yield performance under drought conditions. The aim of this work was to perform a QTL analysis of malting quality traits in a doubled haploid (DH) mapping population of two elite barley lines that differ in their reaction pattern to drought stress.

**Results:**

A population of DH lines was developed by crossing two drought-tolerant elite breeding lines, Victoriana and Sofiara, exploiting distinct mechanism of drought tolerance, sustaining assimilation vs remobilization. The mapping population was assayed under field conditions at four distinct locations that differed in precipitation rate. DH lines were genotyped with the Illumina 9 K iSelect assay, and linkage map including 1782 polymorphic markers and covering a total map length of 1140 cM was constructed. The result of quantitative trait loci (QTL) analysis showed that majority of the traits were affected by several main effect QTL and/or QTL x environment (QE) interactions. In total, 57, 41, and 5 QTL were associated with yield-related traits, malting quality traits and seed quality traits, respectively. 11 and 29 of mapped QTL explained more than 10 and 5% of phenotypic variation, respectively. In several chromosomal regions co-localization between QTL for various traits were observed. The largest clusters were detected on chromosomes 3H and 4H.

**Conclusions:**

Our QTL mapping results revealed several novel consistent genomic regions controlling malting quality which could be exploited in marker assisted selection. In this context, the complex QTL region on chromosome 3H seems of particular interest, as it harbors several large effect QTL.

**Electronic supplementary material:**

The online version of this article (10.1186/s12870-018-1323-4) contains supplementary material, which is available to authorized users.

## Background

Barley (*Hordeum vulgare*) is an important crop cultivated throughout the world with Russia, France, Germany, Australia and Canada being the major producers. It is used as human food, livestock feed and for malt products. About 30% of the global barley production (133.5 million tons) is processed as malt by the brewing or the distilling industry [1, 2]. Therefore, one of the primary breeding goals is to improve the malting quality.

Malting quality is the result of complex interactions of numerous trait components, each of which is under control of multiple genes [3, 4]. Therefore, phenotypic selection of malting quality is labor-intensive and costly [5]. Marker-assisted selection is a promising alternative to improve malting quality [6] but necessitates detailed knowledge of its genetic architecture. Several linkage and association mapping studies have been conducted and QTL controlling components of barley malting quality, such as alpha- and beta-amylase activity, malt extract, malt soluble nitrogen, diastatic power, Kolbach index, wort β-glucans, wort viscosity, friability and apparent final attenuation have been identified [7–12]. Malting quality QTL were mapped to all seven barley chromosomes, however, the distribution was uneven and in some cases the formation of QTL clusters simultaneously affecting several malting traits have been observed [13–15]. Moreover, detected QTL were frequently dependent on genetic background and environmental conditions [16–18].

A few QTL explaining a large proportion of the genotypic variance have been used in marker-assisted selection (MAS). Igartua et al. [19] applied MAS for two QTL regions on chromosome 5H affecting several malting quality traits in the Harrington/TR306 population. Selection for the Harrington allele at target regions produced DH lines with superior malting quality characteristics such as low grain protein and β-glucan content, high diastatic power and malt extract. Ayoub et al. [20] introgressed a QTL from chromosome 5H affecting alpha-amylase activity originating from the cultivar Morex into the feed barley cultivar Labelle and observed an increase in alpha-amylase activity. Laido et al. [21] reported successful marker-assisted introgression of major QTL regions for grain protein content, malt extract, friability and viscosity on chromosome 1H in a population consisting of DH lines derived from winter and spring barley cross (Nure/Tremois). MAS for malting quality QTL may greatly benefit the introduction of novel diversity into elite germplasm. This is of particular importance if complex traits such as tolerance to biotic and abiotic stresses are to be introgressed from unadapted germplasm sources.

Previously, we have identified a number of barley lines showing senescence or stay-green phenotype and demonstrating the superior yield performance under drought conditions. The stay-green and senescing lines differed in their assimilation performance under drought stress. Moreover, it was revealed that senescing lines synthesized greater levels of ABA than the stay-green lines under short-term stress, and it continued to maintain a high level of ABA and ABA catabolites under long-term stress. This suggests that a greater flux in ABA metabolism in the senescing lines negatively affected water use efficiency and assimilation [22]. The objective of the current study was to perform a QTL analysis of malting quality traits in a biparental mapping population of two elite barley lines that differ in their reaction pattern to drought stress in order to identify genetic regions associated with twenty two malting-, grain-quality and yield-related traits.

## Results

### Phenotypic analysis

The BLUEs of the DH population were widely distributed for all examined traits approximating in most cases a normal distribution (Additional file 1: Figure S1; Table 1). For all traits we observed transgressive variation with several DH lines possessing phenotypic values lower or higher than their parental lines. The genotypic variance components$\Big({\sigma}_G^2$) were for all traits except seed nitrogen content (SNC) significantly (*P* < 0.05) larger than zero (Table 2). For 14 out of the 22 measured traits, significant (P < 0.05) variance components were detected for genotype-by-environment interaction effects $\Big({\sigma}_{G\times E}^2$). The ratio of ${\sigma}_G^2:{\sigma}_{G\times E}^2$ was minimum for grain yield (YLD) amounting to 0.27. The broad-sense heritability estimates (h^2^) ranged from 0.34 for SNC to 0.98 for seed length (SL).

**Table 1:** Summary statistics of the evaluated quantitative traits for the parents and the Sofiara/Victoriana DH population across environments

	MMC	FGE	VIS	PRO	SNI	KOL	FLA	FRI	BGL	TML	REL	RTL	YLD	TGW	SA	SB	SL	SSC	SNC	SCC	FT^a^	PH^a^
Victoriana	5.96	80.60	1.50	11.07	742.72	42.53	80.89	75.73	371.27	9.84	4.97	4.88	54.14	51.89	27.81	4.01	9.18	26.78	1.71	40.66	23.67	68.00
Sofiara	5.82	83.36	1.46	11.22	857.44	47.75	81.53	88.15	152.66	11.18	5.71	5.47	49.32	53.47	28.56	4.19	9.09	31.13	1.72	40.83	25.42	82.84
Maximum	6.10	83.50	1.58	11.77	855.70	50.36	82.46	92.40	683.20	11.93	6.30	5.97	60.20	57.66	30.70	4.32	9.65	31.87	1.73	41.09	25.5	74.00
Minimum	5.40	80.84	1.43	9.80	683.20	39.45	79.54	64.64	88.76	8.78	4.75	3.93	48.11	48.13	25.90	3.94	8.43	25.43	1.48	40.46	20.28	66.00
Mean	5.67	82.43	1.49	10.64	763.50	45.05	80.96	81.42	281.90	10.33	5.37	4.97	54.03	52.36	27.67	4.09	8.96	28.31	1.60	40.85	22.83	70.24
SD	0.17	0.58	0.03	0.43	38.62	2.42	0.72	6.65	144.50	0.52	0.29	0.38	2.07	2.07	0.85	0.07	0.21	1.30	0.05	0.12	1.064	1.78
CV	2.94	0.71	2.23	4.00	5.06	5.37	0.89	8.17	51.28	5.07	5.39	7.64	3.83	3.95	3.07	1.74	2.37	4.60	3.16	0.30	4.662	2.53
Skewness	0.72	−0.45	0.63	0.41	0.21	−0.15	− 0.07	− 0.34	0.95	− 0.19	0.27	0.07	− 0.09	0.36	0.62	0.50	0.46	0.22	0.29	− 0.58	0.0804	− 0.13
Kurtosis	−0.34	− 0.45	− 0.14	− 0.16	− 0.44	− 0.49	− 0.81	− 0.91	− 0.03	0.34	0.55	0.21	0.23	− 0.49	0.65	0.33	0.74	− 0.03	− 0.30	0.28	− 0.08	− 0.34

*MMC* moisture content, *FGE* fine grind extract, *VIS* viscosity, *PRO* protein content, *SNI* soluble nitrogen, *KOL* kolbach index, *FLA* limit of attenuation, *FRI* friability, *BGL* β-glucan, *TML* total malting losses, *PH* plant height, *FT* flowering time, *REL* respiration losses, *RTL* rootlet losses, *TGW* thousand-grain weight, *SA* seed area, *SB* seed breadth, *SL* seed length, *YLD* grain yield, *SSC* seed starch content, *SNC* seed nitrogen content, *SCC* seed carbon content, *SD* standart deviation, *CV* coefficient of variation

^a^traits were measured only in some environments

**Table 2:** Estimates of variance components for measured traits across environments

Trait	Environments	σ^2^G	σ^2^E	σ^2^GxE	σ^2^e	h^2^
MMC	3	0.016***	0.009***	0.026***	0.014	0.569
FGE	3	0.236***	0.100***	0.118***	0.131	0.761
VIS	3	0.001***	0.000***	0.001***	0.001	0.575
PRO	3	0.084***	0.474***	0.231***	0.122	0.433
SNI	3	979.487***	55.790*	941.560***	643.514	0.669
KOL	3	3.695***	5.793***	3.167***	2.720	0.675
FLA	3	0.163*	0.221***	0.508***	0.442	0.363
FRI	3	33.685***	3.004***	19.203***	13.321	0.772
BGL	3	15,979.8***	272.305	9595.9***	6179.561	0.768
TML	3	0.089*	3.887***	0.094	0.233	0.489
REL	3	0.038***	1.171***	0.032**	0.057	0.593
RTL	3	0.046*	0.786***	0.043	0.124	0.492
YLD	6	1.735***	200.854***	6.506***	6.908	0.536
TGW	6	3.687***	7.974***	2.119***	1.655	0.891
SA	4	0.654***	0.778***	0.002	0.126	0.974
SB	4	0.004***	0.009***	0.001***	0.002	0.867
SL	4	0.042***	0.058***	0.000	0.005	0.985
SSC	3	0.856***	8.602***	1.253***	1.588	0.551
SNC	3	0.000	0.016***	0.000	0.003	0.336
SCC	3	0.005*	0.019***	0.006*	0.019	0.449
PH	2	1.311**	102.928***	NA	3.541	0.420
ME	2	1.993***	0.358***	NA	1.019	0.800

σ^2^G - genotypic variance, σ^2^GxE - genotype by environment interaction variance, σ^2^e - error variance, h^2^ - heritability, ***p < 0.001, **p < 0.01, *p < 0.05. Abbreviations of the traits are the same as in Table 1

### Correlation estimates

Pearson correlation coefficients were estimated between all pairs of traits (Table 3). Grain yield was negatively correlated (P < 0.05) with soluble nitrogen. Correlation coefficients between malting quality traits ranged from − 0.92 to 0.86. Seed quality traits such as SNC and seed starch content (SSC) were moderately negatively correlated with fine-grind malt extract (FGE) (*r* = − 0.49) and rootlet losses (RTL) (*r* = − 0.28), respectively.

**Table 3:** Pearson correlation coefficients between measured traits

MMC																						
FGE	−0.10																					
VIS	**0.27**	**−0.45**																				
PRO	0.15	**−0.52**	**0.29**																			
SNI	− 0.03	**0.21**	**− 0.39**	**0.31**																		
KOL	−0.14	**0.57**	**− 0.58**	**− 0.43**	**0.72**																	
FLA	**−0.26**	**0.40**	**− 0.72**	**− 0.35**	**0.23**	**0.48**																
FRI	−0.18	**0.57**	**−0.79**	**−0.28**	**0.54**	**0.72**	**0.65**															
BGL	0.17	**−0.49**	**0.86**	**0.24**	**−0.55**	**− 0.69**	**− 0.67**	**− 0.92**														
TML	−0.07	0.10	**−0.23**	0.07	**0.28**	**0.22**	**0.24**	0.11	−0.19													
REL	−0.17	0.14	−0.17	0.00	**0.29**	**0.28**	0.19	**0.22**	**−0.28**	**0.69**												
RTL	0.03	0.02	−0.18	0.11	0.15	0.07	0.18	−0.02	−0.05	**0.83**	0.17											
YLD	0.15	0.19	0.02	−0.16	**−0.25**	−0.12	−0.07	− 0.08	0.04	0.11	0.02	0.16										
TGW	**0.20**	0.09	0.11	0.02	0.09	0.06	**−0.28**	−0.06	0.06	−0.18	−0.06	**− 0.20**	**0.33**									
SA	0.15	0.01	0.13	0.02	0.15	0.12	**−0.34**	−0.05	0.07	**− 0.35**	**−0.21**	**− 0.31**	0.17	**0.84**								
SB	0.06	**0.23**	−0.01	0.19	**0.23**	0.07	−0.13	0.15	−0.11	− 0.13	− 0.06	−0.12	**0.22**	**0.64**	**0.60**							
SL	0.17	−0.08	0.12	−0.10	0.11	0.17	**−0.29**	−0.09	0.08	**−0.35**	**− 0.23**	**− 0.30**	0.10	**0.65**	**0.88**	0.18						
SSC	**0.07**	**0.24**	−0.05	−0.12	0.13	0.18	0.01	0.13	−0.11	**−0.22**	− 0.03	**−0.28**	0.09	**0.62**	**0.62**	**0.45**	**0.50**					
SNC	−0.07	**−0.49**	0.03	**0.54**	**0.24**	−0.16	−0.13	− 0.14	0.09	0.00	−0.11	0.09	**−0.36**	− 0.06	0.06	0.11	−0.03	− 0.07				
SCC	−0.40	− 0.13	−0.14	**0.20**	0.03	−0.12	0.14	0.06	−0.09	0.06	0.06	0.03	0.03	0.10	0.00	**0.24**	−0.15	0.04	**0.34**			
FT	0.11	−0.07	−0.01	0.02	**−0.26**	**−0.27**	0.07	−0.06	0.07	0.02	−0.07	0.09	0.15	−0.04	−0.15	0.16	**−0.26**	− 0.03	0.02	0.02		
PH	0.03	0.05	0.08	**0.23**	0.07	−0.13	−0.07	0.04	0.04	0.09	0.10	0.04	−0.06	0.11	0.12	**0.30**	−0.06	**0.30**	0.09	0.15	**0.20**	
	MMC	FGE	VIS	PRO	SNI	KOL	FLA	FRI	BGL	TML	REL	RTL	YLD	TGW	SA	SB	SL	SSC	SNC	SCC	FT	PH

Significant correlations (*P* < 0.05) are labeled in bold text. Abbreviations of the traits are the same as in Table 1

### QTL analysis

The 1782 mapped SNP markers detected a total of 380 independent loci. Hence, for QTL mapping 380 polymorphic high-quality SNP markers were selected, i.e. one marker per locus. The number of polymorphic markers ranged from 106 for chromosome 1H to 374 for chromosome 2H (Additional file 2: Table S1). Despite the high marker density, we observed some regions such as on the chromosome 1H exhibiting a low number of polymorphic markers. It is likely that these regions are identical by descent in both parents.

**Fig. 1:**
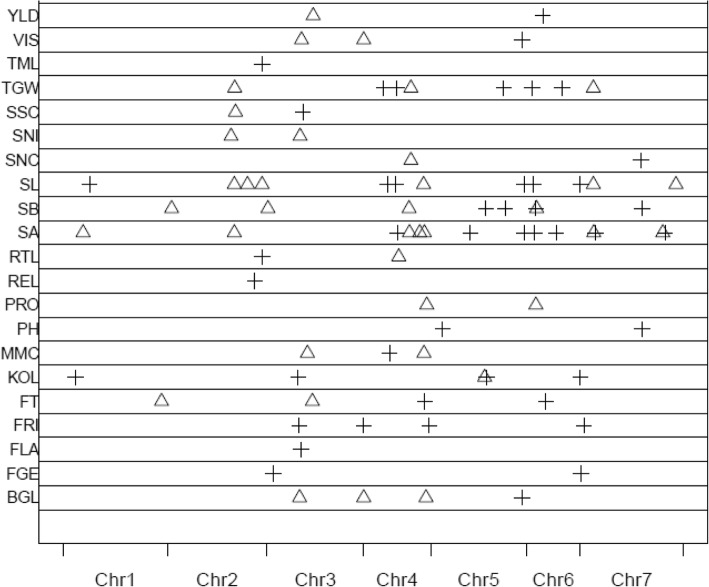
Location of main-effect QTL on chromosomes. Triangle and cross represent favorable QTL alleles contributed by Victoriana and Sofiara, respectively

Genome-wide mapping resulted in 103 QTL with additive main effects and/or additive x environment interaction effects (QE) (Tables 4, 6, Fig. 1). No QTL were detected for seed carbon content. Twenty-nine QTL explained more the 5% and 11 QTL even more than 10% of the phenotypic variation. Variation explained by main effects (QTL) ranged from 0.41 to 68.77%, which was substantially higher than that of QTL x Environment interaction effects.

**Table 4:** Main-effect QTL for measured traits detected in the Sofiara/Victoriana DH population in six environments

Trait	QTL name	Chr.	Position (cM)	Peak Marker	LOD	Effect	R2 (%)
Malt moisture content							
	QMMC-3	3H	74.64	BOPA1_2616–2560	4.27	−0.04	2.66
	QMMC-4-1	4H	47.94	BOPA1_10346–92	6.38	0.04	1.91
	QMMC-4-2	4H	111.57	BOPA1_3652–872	6.52	−0.06	3.67
Fine grind extract							
	QFGE-3	3H	11.55	SCRI_RS_153718	5.84	0.22	6.96
	QFGE-7	7H	1.53	BOPA1_7970–387	4.52	0.21	6.76
Viscosity							
	QVIS-3	3H	63.66	BK_08	13.91	0.00	13.82
	QVIS-4	4H	0.00	BOPA2_12_30540	6.83	−0.01	8.48
	QVIS-5	5H	169.36	BOPA1_1394–1222	5.92	0.01	5.50
Malt protein content							
	QPRO-4	4H	116.80	SCRI_RS_179695	11.75	−0.21	5.06
	QPRO-6	6H	17.05	BOPA1_397–288	7.76	−0.18	4.50
Soluble protein							
	QSNI-2	2H	118.20	BOPA1_11591–265	4.06	−5.85	1.10
	QSNI-3-1	3H	60.96	BK_08	25.79	−68.76	27.31
Kolbach index							
	QKOL-1	1H	21.79	SCRI_RS_205669	5.80	0.41	0.85
	QKOL-3	3H	57.11	SCRI_RS_115045	9.93	0.17	13.74
	QKOL-5-1	5H	100.05	BOPA2_12_31361	4.70	−2.04	1.34
	QKOL-5-2	5H	103.41	BOPA1_370–443	4.26	1.68	1.48
	QKOL-7	7H	0.00	BOPA1_7970–387	8.00	0.94	4.05
Limit of attenuation							
	QFLA-3	3H	62.76	BK_08	5.26	0.32	6.85
Friability							
	QFRI-3	3H	59.08	SCRI_RS_115045	36.80	4.40	33.81
	QFRI-4-1	4H	0.00	BOPA2_12_30540	5.42	1.64	3.93
	QFRI-4-2	4H	121.34	BOPA1_ABC08009–1–2-304	7.04	1.80	4.68
	QFRI-7	7H	6.80	BOPA2_12_31173	8.94	2.29	6.64
β-glucan							
	QBGL-3	3H	60.06	BK_08	38.62	−81.46	37.42
	QBGL-4-1	4H	0.00	BOPA2_12_30540	6.59	−32.38	4.19
	QBGL-4-2	4H	115.31	BOPA1_4160–1365	6.83	−22.12	2.62
	QBGL-5	5H	169.36	BOPA1_1394–1222	6.01	38.15	4.29
Total malting losses							
	QTML-2	2H	175.69	BOPA1_8586–1221	5.83	0.25	1.89
Respiration losses							
	QREL-2	2H	162.13	SCRI_RS_193100	8.13	0.16	2.10
Rootlet losses							
	QRTL-2	2H	175.69	BOPA1_8586–1221	5.84	0.13	1.88
	QRTL-4	4H	64.90	SCRI_RS_89959	4.09	−0.07	0.57
Grain yield							
	QYLD-3-1	3H	85.25	SCRI_RS_156056	3.87	−0.88	0.24
	QYLD-6	6H	30.19	BOPA1_2294–573	5.85	0.80	0.32
TGW							
	QTGW-2	2H	124.98	SCRI_RS_209622	7.81	− 0.66	3.39
	QTGW-4-1	4H	36.84	BOPA2_12_30328	5.68	0.44	3.41
	QTGW-4-2	4H	60.73	SCRI_RS_134953	4.30	0.65	0.99
	QTGW-4-3	4H	87.41	12_31523	7.42	−0.84	0.90
	QTGW-5-1	5H	135.55	SCRI_RS_119308	4.67	0.54	5.16
	QTGW-5-2	5H	169.36	BOPA1_1394–1222	4.04	0.48	1.17
	QTGW-6-1	6H	9.93	BOPA2_12_30665	8.03	0.43	3.77
	QTGW-6-2	6H	65.50	BOPA1_3349–759	3.95	0.35	0.94
	QTGW-7	7H	24.27	BOPA1_3187–1073	10.49	−0.43	4.89
Seed area							
	QSA-1-1	1H	35.65	SCRI_RS_117492	4.74	−0.80	0.17
	QSA-2	2H	124.23	SCRI_RS_209622	26.94	−0.53	15.87
	QSA-4-1	4H	62.51	SCRI_RS_140349	7.04	0.37	3.86
	QSA-4-2	4H	84.71	BOPA2_12_31523	4.56	−0.23	0.77
	QSA-4-3	4H	103.70	BOPA2_12_30158	6.86	−0.13	1.27
	QSA-4-4	4H	112.31	SCRI_RS_148330	7.18	−0.06	3.31
	QSA-5-1	5H	73.55	SCRI_RS_205235	4.90	0.15	1.56
	QSA-5-2	5H	173.16	SCRI_RS_102414	21.59	0.23	4.30
	QSA-6-1	6H	14.38	BOPA1_397–288	4.07	0.10	1.16
	QSA-6-2	6H	54.57	BOPA2_12_30698	4.07	0.09	0.31
	QSA-7-1	7H	25.02	SCRI_RS_223021	7.80	−0.45	2.37
	QSA-7-2	7H	28.05	BOPA1_8365–454	5.45	0.28	0.59
	QSA-7-3	7H	152.86	BOPA1_3900–611	4.77	−0.03	0.25
	QSA-7-4	7H	157.25	SCRI_RS_13570	8.69	0.16	0.38
Seed breadth							
	QSB-2	2H	8.13	SCRI_RS_188511	8.33	−0.02	3.75
	QSB-3	3H	1.49	SCRI_RS_155475	5.03	−0.02	2.31
	QSB-4	4H	83.95	BOPA2_12_31246	5.07	−0.01	0.86
	QSB-5-1	5H	102.48	BOPA1_370–443	9.93	0.03	5.31
	QSB-5-2	5H	138.29	SCRI_RS_178985	13.44	0.02	8.58
	QSB-6-1	6H	17.05	BOPA1_397–288	8.56	0.07	1.97
	QSB-6-2	6H	18.55	SCRI_RS_136658	6.77	−0.05	0.83
	QSB-7	7H	114.27	SCRI_RS_194841	4.71	0.02	1.49
Seed length							
	QSL-1	1H	48.06	SCRI_RS_128285	18.98	0.06	1.82
	QSL-2-1	2H	124.23	SCRI_RS_209622	41.34	−0.10	18.13
	QSL-2-2	2H	148.64	SCRI_RS_12444	4.34	−0.04	0.56
	QSL-2-3	2H	175.69	BOPA1_8586–1221	17.56	−0.06	4.57
	QSL-4-1	4H	44.21	BOPA2_12_30597	16.19	0.06	6.09
	QSL-4-2	4H	59.83	SCRI_RS_134953	6.89	0.03	0.56
	QSL-4-3	4H	110.82	12_30987	19.91	−0.08	3.30
	QSL-5	5H	173.16	SCRI_RS_102414	19.46	0.05	2.14
	QSL-6	6H	12.60	BOPA2_12_30665	10.02	0.04	1.15
	QSL-7-1	7H	0.00	BOPA1_7970–387	8.08	0.05	1.10
	QSL-7-2	7H	24.27	BOPA1_3187–1073	16.42	−0.08	3.00
	QSL-7-3	7H	177.23	BOPA1_6470–1005	5.17	−0.02	0.29
Seed starch content							
	QSSC-2	2H	126.48	BOPA2_12_10735	4.10	−0.50	2.39
	QSSC-3	3H	66.37	SCRI_RS_168665	3.96	0.41	1.69
Seed nitrogen content							
	QSNC-4-1	4H	87.41	BOPA2_12_31523	5.33	−0.03	2.77
	QSNC-7	7H	112.75	SCRI_RS_104566	4.39	0.02	2.17
Flowering time							
	QFT-1	1H	180.94	SCRI_RS_196025	4.60	−0.37	12.99
	QFT-3	3H	83.76	BOPA1_4150–398	6.47	−0.48	12.90
	QFT-4	4H	113.06	SCRI_RS_217794	5.29	0.35	8.45
	QFT-6	6H	35.43	BOPA1_4146–1154	4.27	0.21	3.63
Plant height							
	QPH-5	5H	22.40	BOPA1_6184–200	4.82	0.44	0.37
	QPH-7	7H	114.27	SCRI_RS_194841	4.18	0.59	0.50

### QTL and environment interactions for yield-related traits

The QTL analysis identified a total of 50 QTL for five yield-related traits. Seed area (SA) yielded the highest number of QTL (14) with phenotypic variation in the range of 0.17 to 15.87%. QTL of highest LOD score for SA were mapped on chromosome 2H and 5H explaining 15.87 and 4% of phenotypic variation, respectively. One significant environmental interaction effect was found for QSA-1-2. Twelve main effect QTL were observed for SL. The phenotypic variation explained by a single QTL ranged from 0.29 to 18.13%. A major QTL with a LOD value of 41.3 was found on chromosome 2H near marker SCRI_RS_209622. None of the QTL showed significant QE interaction. Thousand grain weight (TGW) and seed breadth (SB) were also among the traits with the highest number of QTL, 9 and 8 respectively. Loci affecting TGW were found to be located on chromosomes 2H, 4H, 5H, 6H and 7H and phenotypic variance explained by individual QTL ranged from 0.90 to 5.16%. Two QTL, QTGW-3-1 and QTGW-3-2 displayed significant QE interactions. QTL for SB were located on each chromosome except 1H and explained from 0.83 to 8.58% of the phenotypic variation. QSB-3 had only significant QE interaction effect. Four significant QTL affecting YLD were detected on three chromosomes (3H, 4H and 6H) and collectively explained 1.27% of phenotypic variation (Additional file 3: Table S2). QYLD-3-2 and QYLD-4 showed QE interaction only whereas QYLD-3-1 and QYLD-6 showed main effects only. The alleles for higher yield of QYLD-3-1 and QYLD-6 were contributed by Victoriana and Sofiara, respectively.

### QTL for malting quality-related traits

Overall, 41 significant QTL were identified for twelve malting quality traits, ranging from 1 to 11 QTL per trait. There were five QTL that explained more than 10% of phenotypic variance for such traits as wort viscosity (VIS), soluble nitrogen (SNI), Kolbach Index (KOL), friability (FRI) and wort β-glucan content (BGL). Among all traits, the highest numbers of main effect QTL were detected for KOL (5), which were mapped to four chromosomes. The highest number of QE interaction effects were detected for SNI (9), located to three chromosomes.

Fourteen QTL with phenotypic variation in the range of 0.62 to 27.31% were detected for SNI. They were mapped to four chromosomes (2H, 3H, 4H and 5H). There were two main-effect QTL and nine QE interaction effects which did not show significant main effect. The proportion of collective phenotypic variation explained by main-effects and QE interaction effects were 33.44 and 13.06% (Additional file 3: Table S2). This suggests that the environmental interaction effects were important for the expression of SNI. The phenotypic effects of QE demonstrated large variation across the environments both in the magnitude and direction. The largest main effect QTL (QSNI-3-1) was mapped on chromosome 3H at 60.96 cM near the marker BK_08. The superior allele was contributed by Sofiara and explained 27.31% of the phenotypic variation with a LOD of 25.79.

For KOL six QTL were identified, explaining 0.85 to 13.74% of the phenotypic variance. At five loci the Sofiara alleles had increasing effects on the trait and only at one (QKOL-5-1) caused the reduction. Only single QTL located on chromosome 3H (QKOL-3) with significant main effect showed QE interactions.

A total of 6 QTL located on four chromosomal regions 2H, 3H, 4H and 5H were associated with BGL. Collectively, these four main effect QTL and QE interactions explained 50.80 and 11.99% of the phenotypic variation, respectively. Alleles for the QBGL-3, QBGL-4-1 and QBGL-4-2 for increased BGL content were contributed by Victoriana and at QBGL-5 the Sofiara allele increased the trait value. The major QTL, explaining up to 37.42% of phenotypic variation in BGL was located on 3H chromosome at 60.06 cM.

Four loci controlling FRI were identified on three chromosomes. The phenotypic variance explained by each QTL ranged from 3.93 to 33.81%. In all cases the alleles responsible for increasing the trait value were contributed by Sofiara. Collectively, the four main effect QTL explained 50.78% of the phenotypic variation. The largest QTL (QFRI-3) with LOD value 36.80 was identified on chromosome 3H at position 59.08 cM and explained 33.81% of the phenotypic variance. QFRI-3 also showed QE effect that explained 12.41% of the phenotypic variation.

Two main effect QTL were identified for malt protein content (PRO). The QTL were located on chromosomes 4H and 6H and explained 5.1 and 4.5% of the phenotypic variation, respectively. The Victoriana allele at each locus increased the protein content.

Four QTL for VIS were identified on three chromosomes. The phenotypic variance explained by each QTL ranged from 4.57 to 13.82%. Collectively, three main effect QTL and one QE interaction effect explained 29.25 and 1.67% of the phenotypic variation, respectively. In most cases the Victoriana alleles were responsible for increased trait value. The largest QTL (QVIS-3) with LOD value 13.91 was located on chromosome 3H at position 63.66 cM and explained 13.82% of the phenotypic variance.

For FGE, a total of three QTL were detected that mapped to chromosomes 1H, 3H and 7H. QFGE-3 and QFGE-7, which were main effect QTL collectively explained 21.37% of the phenotypic variation and positives alleles at these loci were contributed by Sofiara. One QE interaction effect (QFGE-1) was detected on chromosome 1H and explained 0.99% of phenotypic variance.

Three main effect QTL were detected for MMC. One was located on chromosome 3H at position 74.64 cM and explained 2.66% of the phenotypic variation. The other two were mapped on chromosome 4H at position 47.94 and 111.57 cM and explained 1.9 and 3.67% of the phenotypic variance, respectively. At the locus QMMC-3 and QMMC-4-2 positive effects were associated with Victoriana alleles and at QMMC-4-1 the Sofiara allele increased the trait value.

Three main effect QTL affecting malting losses were identified. Two QTL for RTL were located on chromosome 2H and 4H and one QTL for respiration losses (REL) on chromosome 2H. The percentage of phenotypic variation explained by individual QTL varied between 0.57 and 2.1%. The positive alleles for the QREL-2 and QRTL-2 were contributed by Sofiara and for QRTL-4 by Victoriana.

Only one QTL was detected for limit of attenuation (FLA) with LOD value 5.26. It was located on chromosome 3H near the marker BK_08 and explained 6.85% of phenotypic variance. The Sofiara allele contributed to the increased trait value.

### QTL for seed-quality traits

A total of five significant QTL located on four chromosomes (2H, 3H, 4H and 7H) were detected for three seed-quality traits (Tables 4, 6, Fig. 1). The percentage of phenotypic variation explained by these QTL varied from 1.69 to 2.77%. For SSC, two main effect QTL (QSSC-2 and QSSC-3) were detected. The first QTL was located at the position 126.5 cM on 2H, explaining of the 2.38% of phenotypic variation. The second one was found on 3H at the position 66.4 cM and explained the 1.69% of the phenotypic variance. No significant QE interaction was detected for SSC. Two main effect QTL influencing SNC were observed on the chromosome 4H and 7H at the position 87.4 and 112.7 cM, respectively. These QTL accounted for 2.17–2.77% of the phenotypic variation in the trait. QTL on chromosome 4H (QSNC-4-2) showed no significant main effect while QE interaction effect was detected, explaining 2.01% of the phenotypic variance.

### QTL for plant height and flowering time

Three QTL for plant height (PH) were found. QPH-5 explained 0.37% of variance. QPH-7 was located on 7H at position 114.27 cM near the marker SCRI_RS_194841 explaining 0.5% of phenotypic variation. QE interaction effect (QPH-2) was identified on chromosome 2H and explained 1.09% of phenotypic variation.

Four main effect QTL for flowering time (FT) with an LOD > 3 that collectively accounted for 46.66% of phenotypic variance were mapped. Victoriana alleles increased the time to flowering at the QFT-1 and QFT-3 and reduced it at the QFT-4 and QFT-6.

### Co-localizations of QTL

For several traits hot spots of co-locating QTL were observed on all chromosomes except for 1H. It is well known that such clustering is a consequence of either close linkage or pleiotropy. The highest level of clustering occurred in two regions: in the centromeric region of chromosome 3H and at the bottom of chromosome 4H (Additional file 4: Figure S2). The cluster on chromosome 3H mainly comprises malting quality QTL for BGL, FRI, KOL, VIS and SNI (R^2^ > 10%). The cluster on 4H contained QTL for BGL, FRI and PRO together with several QTL for seed morphological traits. QTL for BGL were found to be most often co-localized with loci affecting FRI and VIS. These traits were highly correlated to each other (Table 3). A higher content of one component (BGL/VIS) corresponded to a lower content of the other (FRI). This was corroborated by the observed allelic effects, with Victoriana alleles increasing BGL content and decreasing FRI. In two chromosomal regions (3H, 4H) QTL for BGL co-localized with loci affecting FRI and VIS. In two other regions on chromosome 4H and 5H BGL QTL coincided with QTL for FRI or VIS, respectively. QBGL-3 co-localized not only with QTL for VIS and FRI but also with loci affecting FLA, SSC and SNI. The region involved in BGL variation in the distal region of chromosome 4H also accounted for variation in the protein content. Here, the Victoriana allele caused an increase in BGL and PRO content (Table. 5). Protein and β-glucan metabolism are independent biological processes therefore the co-localisation of QTL in this case is likely due to linkage between different genes than due to pleiotropy. Clusters of QTL affecting yield-related traits such as TGW, SL, SB and SA were observed on chromosomes 2H, 4H, 5H, 6H and 7H. The QTL cluster on chromosome 2H was also associated with SSC and SNI, while the cluster on the chromosome 5H involved QTL influencing BGL and VIS. We also observed that two regions on chromosomes 3H and 6H harbor coinciding QTL for YLD and FT, reflecting the interaction between these traits. The positive alleles for both traits at the cluster on chromosome 3H and 4H were derived from Victoriana and Sofiara, respectively.

**Table 5:** QTL x Environment interaction effect for measured traits detected in the Sofiara/Victoriana DH population in six environments

Trait	QTL name	Chr.	Position (cM)	Nearest Marker	LOD	R^2^ (%)^a^	E1	E2	E3	E4	E5	E6
Fine grind extract												
	QFGE-1	1H	180.94	SCRI_RS_196025	5.08	4.39	−0.019	0.242	NA	−0.148	NA	NA
Viscosity												
	QVIS-3	3H	60.06	BK_08	5.17	4.57	−0.008	−0.006	NA	−0.027	NA	NA
Soluble protein												
	QSNI-3-2	3H	60.06	BK_08	12.85	9.09	82.810	69.669	NA	102.227	NA	NA
	QSNI-3-3	3H	72.37	SCRI_RS_231801	7.52	0.73	17.337	52.080	NA	−11.807	NA	NA
	QSNI-3-4	3H	73.89	BOPA1_2616–2560	7.54	1.86	−15.369	−32.424	NA	20.908	NA	NA
	QSNI-3-5	3H	83.02	SCRI_RS_225522	5.93	0.62	26.691	19.770	NA	2.040	NA	NA
	QSNI-3-6	3H	85.25	SCRI_RS_156056	6.93	0.65	−19.966	−22.262	NA	7.317	NA	NA
	QSNI-4	4H	54.76	BOPA1_ABC08788–1–1-329	4.76	1.90	−7.178	−7.735	NA	7.375	NA	NA
	QSNI-5-1	5H	93.27	SCRI_RS_159611	5.41	3.60	43.891	17.155	NA	20.709	NA	NA
	QSNI-5-2	5H	97.05	SCRI_RS_128664	6.04	2.03	−44.480	9.496	NA	12.656	NA	NA
	QSNI-5-3	5H	98.54	BOPA1_ABC14990–1–1-126	5.75	1.73	−1.879	−30.265	NA	−22.775	NA	NA
Kolbach index												
	QKOL-3	3H	60.06	BK_08	9.28	5.63	1.158	0.485	NA	2.630	NA	NA
Friability												
	QFRI-3	3H	59.08	SCRI_RS_115045	19.48	11.36	2.622	9.418	NA	4.398	NA	NA
β-glucan												
	QBGL-2	2H	147.15	SCRI_RS_156220	4.93	3.27	−3.787	3.058	NA	−57.402	NA	NA
	QBGL-3	3H	60.06	BK_08	22.93	12.70	−81.459	−46.583	NA	−190.728	NA	NA
Grain yield												
	QYLD-3-2	3H	71.61	SCRI_RS_231801	6.39	0.37	1.521	0.302	0.607	0.126	−1.331	−0.322
	QYLD-4	4H	109.33	BOPA1_1241–1649	7.73	0.47	0.696	0.210	−1.231	−0.932	1.241	0.691
TGW												
	QTGW-3-1	3H	60.06	BK_08	8.17	1.64	0.559	0.228	−0.147	0.359	−0.562	0.157
	QTGW-3-2	3H	79.25	BOPA1_10317–448	6.43	0.22	0.011	0.340	−0.227	0.016	−0.443	−0.014
Seed area												
	QSA-1-2	1H	37.44	SCRI_RS_128285	5.64	1.59	0.741	0.749	1.053	0.874	NA	NA
Seed breadth												
	QSB-3	3H	72.37	SCRI_RS_231801	5.70	1.68	−0.012	−0.002	−0.008	0.030	NA	NA
Seed nitrogen content												
	QSNC-4-2	4H	81.71	SCRI_RS_238618	4.89	2.01	−0.011	−0.006	NA	0.030	NA	NA
Plant height												
	QPH-2	2H	124.23	SCRI_RS_209622	9.00	1.09	NA	NA	−0.380	1.060	NA	NA

E1- well watered Gatersleben 2012, Germany; E2- drought stress Gatersleben 2012, Germany; E3- rainfed Wohlde 2011, Germany; E4- rainfed Wohlde 2012, Germany; E5- rainfed Walewice 2011, Poland; E6- rainfed Walewice 2012, Poland

^a^R^2^ collective percentage of phenotypic variation explained by QE interactions; NA - not analysed

## Discussion

In the present study comprehensive phenotyping, genotyping and QTL analysis of a population derived from two elite barley lines, that differ for drought tolerance behavior, has led to the identification of numerous QTL for yield and yield components as well as for seed and malting quality. On all chromosomes except 1H the hot spots carrying QTL for three or more traits where identified.

### Main effect QTL

Our analysis resulted in identification of 85 main effect QTL for 21 traits. Victoriana alleles increased the trait value in 36 instances, while in 49 cases they were responsible for decreasing of trait expression. For about half of the traits parental alleles of both increasing and decreasing effects were observed. This underscores on the one hand the complex inheritance and on the other hand the transgressive potential of the cross. However, for two traits (PRO and SNI) the increased trait performance was associated with Victoriana alleles; while only Sofiara alleles were responsible for increasing of the trait value for FGE, FRI, FLA, total malting losses (TML), REL and PH. We will focus on QTL which are supposed important to understand the genetic architecture of a given trait and could be useful for further improvement of malting quality.

TGW is important yield component, and many investigations to determine genomic regions controlling this trait have been performed. QTL for TGW were mapped across all seven chromosomes in various bi-parental populations and diverse panels for association mapping studies [23–26]. In our study, a total of nine QTL were detected for TGW. As expected, high positive correlations with r values in the range of 0.64 to 0.84 were observed between TGW and SL, SB and SA confirming that TGW is mainly a function of seed volume. Indeed, co-localization of each QTL for TGW and at least one of the parameters for seed volume were observed. With regard to potential improving of TGW, the two QTL localized on long arm of 5H and short arm of 7H were of special interest, because they explained about 5% of phenotypic variation. The alleles for increasing TGW at QTGW-5-1 and QTGW-7 were contributed by Sofiara and Victoriana, respectively. The QTGW-5-1 and QTGW-7 were found to correspond to QTL detected previously by Rode et al. [25] and Schnaithmann and Pillen [26], respectively; whereas QTGW-5-2, corresponding to the marker BOPA1_1394–1222 at the peak position 169.4 cM is a newly identified locus. QTGW-6-1 and QTGW-6-2, found in our study, were both derived from Sofiara. They coincided with previously reported QTL for TGW [26], suggesting that these genetic factor(s) influencing TGW could operate in a different genetic background. QTGW-6-2 could be considered of special interest, as it was previously reported that loci at this position were able to increase TGW under nitrogen deficiency [26].

A total of 34 main effect QTL were determined for seed morphological traits. A half (50%) of QTL was contributed by Sofiara alleles while a second half by Victoriana. QSL-1 was coincident with QSA-1-1 and found to correspond to the locus controlling seed length detected by Kalladan et al. [27]. In the same genomic region the gene encoding drought-responsive MYB transcription factor was mapped [28]. The largest QTL, explaining more than 5% of phenotypic variation, were mapped on chromosomes 2H, 4H and 5H. QSL-2-1, mapped to the distal part of chromosome 2H at the marker SCRI_RS_209622 co-localized with QSA-2 and QTGW-2. For all these QTL the alleles increasing the trait value were contributed by Victoriana. That could suggest the presence of the loci with pleiotropic effect in this genomic region. Of interest to note that no QTL for SL were found in this region on chromosome 2H using the set of barley introgression lines S42ILs, produced from the cross Scarlett/ISR42–8. QTL affecting SA was identified on chromosome 2H, however, it was associated with different genomic region [26]. Kalladan et al. [27] recently reported mapping QTL for SL and TGW on chromosome 2H in the DH population Brenda/HS584. Due to absence of common markers, it is difficult to conclude whether they match to the ones identified in our study. However, a previously identified QTL for SL mapped before [27] was assigned to bin 8 according to the BinMap2005 (http://wheat.pw.usda.gov/cgi-bin/cmap), while QSL-2-1 detected in our study belongs to the bin 11. Therefore it could be considered as a newly identified locus. In addition, we have identified several QTL affecting such morphological seed traits as SL, SB and SA on chromosome 4H. QTL for SL mapped on chromosome 4H were not identified in previous studies, either in the S42IL or Brenda/HS584 populations. Therefore they could be considered as newly identified loci. QTL for SB and SA on chromosome 4H have been identified in the QTL study of wild barley introgression lines S42ILs. Most likely two loci (QSB-4 and QSA-4-1) identified in our study are identical to the ones detected by Schnaithmann and Pillen [26]. However, the additional loci controlling SA (QSA-4-2, QSA-4-3 and QSA-4-4) that all were contributed by Victoriana and have been mapped to the distal part of chromosome 4H in our investigation, represent novel QTL. On chromosome 5H, five loci influencing variation of all three examined seeds morphological traits were mapped. Positive alleles were exclusively contributed by Sofiara. Two of them, QSA5–2 and QSL-5, were mapped to identical position. Recently a locus controlling variation in SL was mapped on chromosome 5H [27], however, its position differs from the one detected in our study.

Our analysis revealed 30 main effect malting quality QTL, explaining 0.6–37.4% of the phenotypic variation. Seventeen favorable QTL alleles were contributed by Sofiara and 13 by Victoriana. 56.7% of those QTL were mapped to chromosomes 3H (8 loci) and 4H (9 loci). Two clusters comprising the largest effect QTL associated with 10 different malting quality traits have been identified on these chromosomes. This finding indicates the importance of these chromosomal regions for developing superior malting phenotype.

### Malt extract and fermentability

In previous mapping studies, QTL for malt extract have been mapped on all seven chromosomes [15, 21, 29, 30]. FGE in the present population was controlled by two main effect QTL positioned on the short arm of 3H and 7H with the favorable alleles of Sofiara, accounting for 7% of the phenotypic variation. Malt extract QTL on the chromosome 3H was reported by Mohammadi et al. [11]. However, that locus was assigned to the long arm of chromosome 3H (bin 16) and could not be matched with QFGE-3 detected in our study. Position of QFGE-7 did not coincide with the localization of the loci controlling malt extract on chromosome7H reported earlier either [1, 13]. However, QFGE-7 was situated in proximity (about 15 cM) to QTL detected by Wang et al. [15] in the TX9425/Naso Nijo population. Comparison based on marker bins (BinMap2005) revealed that QFGE-7 was assigned to the bin 1 while the one detected by Wang et al. [15] to the bin 2 on chromosome 7H. This finding suggests that the top of chromosome 7 contains genetic factors affecting malt extract which operate in different genetic backgrounds.

Regarding the malt wort fermentability, a single QTL for FLA has been localized on the short arm of chromosome 3H at the marker BK_08 (bin 6). Loci influencing fermentability were reported on chromosomes 4H and 6H [31]. In addition, Swanston et al. [12] mapped QTL for fermentability on 3H at the sdw1 locus (bin11) which was suggested to be determined by ß-amylase activity. QFLA-3 detected in our study was contributed by Sofiara and coincided with fermentability locus detected in the Triumph/Morex population by Elía et al. [10]. Our correlation analyses (Table 3) suggested that a number of traits like PRO, FGE, VIS, FRI and BGL influenced FLA, with VIS having the strongest effect. Moreover, QTL for FRI, BGL, SNI, and VIS co-localized to the same genomic region, providing evidence that extent of endosperm modification and wort viscosity are major factors influencing fermentability.

### Viskosity, friability and ß-glucan content

Such traits as VIS, FRI and BGL showed a high correlation to each other. Co-localizations QTL for at least two of these traits were observed on 3H, 4H and 5H. The favorable alleles for FRI QTL were exclusively contributed by Sofiara that was opposed to the loci controlling VIS and BGL which in their majority have been provided by Victoriana. Observed co-localization of loci affecting FRI and VIS could be explained by strong dependence of both traits on the process of degradation of ß-glucans that represent a major component of endosperm cell walls in barley [32]. The BGL QTL with the largest effect has been mapped near the centromeric region of chromosome 3H at the marker BK_08. QBGL-3 matched with QTL for malt ß-glucan reported by Han et al. [33] and was located in close proximity to the region affecting ß-glucan in barley grains [34]. QBGL-4-1 was found to be located in the same region as a QTL for malt ß-glucan reported earlier [33] while the location of QBGL-4-2 and QBGL-5 did not match with positions of the loci controlling BGL detected either in linkage or association mapping [34, 35]. None of mapped BGL QTL coincide with the position of known genes Glb1 and Glb2 on chromosome 1H and 7H, encoding (1,3;1,4)-β-glucanases. This could be explained by lack of genetic differences between the parents at these particular loci. Two loci QVIS-3, QVIS-5, controlling more than 5% of variability in viscosity, were found to match the QTL reported before [15, 21, 31]. In addition we have identified QTL for VIS at the short arm of the chromosome 4H linked to the marker BOPA2_12_30540. Previously, a locus controlling viscosity positioned on chromosome 4H was reported in the VB9524/ND11231_12 population [9], however, its precise location could not be clarified due to lack of common markers in our studies.

A total of four QTL were detected for FRI which were all derived from Sofiara, the parent possessing the favorable alleles (higher trait value). Two of them, QFRI-4-1 and QFRI-7, could be matched to the ones reported before [31, 36, 37]. However, the FRI in our population was mainly influenced by the locus on chromosome 3H which explained 33.8% of genetic variance. QFRI-3 belongs to the bin 6 and was found to be located in close proximity to FRI locus mapped on 3H (bin7) in the population Nure/Tremois [21]. Another interesting novel QTL for increased FRI maps at a distal position of chromosome 4 near marker BOPA1_ABC08009–1–2-304 and accounted for 5% of the phenotypic variation. QFRI-4-2 coincided with QBGL-4-2 and QPRO-4, suggesting that terminal region of chromosome 4H contains closely linked or pleiotropic genes affecting a range of important malting quality traits.

### Protein content, soluble nitrogen, Kohlbach index

Nine QTL were found to be associated with PRO, SNI and KOL, traits that are used for characterization of nitrogen fractions of malt. A major QTL for SNI (QSNI-3-1) was mapped on chromosome 3H near the marker BK_08 in the same chromosomal region where locus controlling variation of SNI in Harrington/Mikamo Golden population was reported by Zhou et al. [38]. A novel QTL for SNI was detected on 2H near the marker BOPA1_11591–265. This QTL explained 1% of the phenotypic variation and was found to be co-located with loci affecting TGW, SA and SL. Genomic regions affecting malt protein content are known on all seven chromosomes [15, 38, 39]. However, two QTL QPRO-4 and QPRO-6 detected in the present study could not be matched with any previous findings. A total of five QTL were detected for KOL, and at four loci the increased KOL was contributed by Sofiara. Locations of QKOL-5-1, QKOL-5-2 and QKOL-7 coincide with position for the loci reported previously [15, 16]. The major QTL, QKOL-3, was mapped on 3H with closest marker SCRI_RS_115045 (bin 6). Locus controlling variation in KOL on chromosome 3H was reported in population 88Ab536/Morex [29]. This QTL was linked with the marker 1_1328 associated with bin 14 and therefore could not be matched with the one identified in current study. In addition, a minor QTL for KOL was mapped on 1H near the marker SCRI_RS_205669 (bin 5). In a similar region, a QTL affecting KOL was reported in wild barley introgression lines S42ILs [14]. In particular, line S24IL-102 bearing an introgression of about 85 cM in size showed a strong reduction of KOL up to 11.5%. However, it has to be noted that the large size of introgressed region covering nine bins obviates a precise comparison of QTL.

### Seed starch and seed nitrogen content

QSNC-4-1 and QSNC-7 were found to match the QTL for grain protein content or N% reported before in the Vlamingh/Buloke and Brenda/HS584 populations, respectively [27, 30]. Locus controlling seed starch content on chromosome 3H (QSSC-3) was co-localized with QTL for a range of important malting quality traits like KOL, FRI, SNI and VIS. It coincides with QTL controlling SSC in the same region reported before by Kalladan et al. [27]. That suggests that the short arm of chromosome 3H contains genetic factors influencing a number of malting quality characters which stay active in different genetic backgrounds and are of primary importance for breeders. In addition, we have mapped QTL for SSC on 2H chromosome contributed by Victoriana. It was linked to the marker BOPA2_12_10735 (bin 11) and could not be matched to any QTL detected in any of above mentioned studies. The same region harbors a locus for SNI at which the Victoriana allele causes an increase in SNI. High SNI content would be considered a disadvantage, because it finally results in decreasing of malt extract. Moreover, QSSC-2 co-localized with QTL affecting yield-related parameters such as SA, SL and TGW. For all three QTL alleles increasing trait value were contributed exclusively by Victoriana.

### QE interaction effects

From the practical point of view it is important to know how the identified loci performed in different environmental conditions. Our analysis revealed that 5 out of 86 main effect QTL showed QE interactions, in particularly QBGL-3, QVIS-3, QFRI-3, QKOL-3 and QSB-3. Phenotypic variation explained by QE interactions amounted to 12.7, 4.6, 11.4, 5.6 and 1.7%, respectively (Table 5). Loci for malting quality traits showed differences only in magnitude, while for QSB-3 differences were observed in both the magnitudes and directions of effects. QBGL-3 showed the largest variation among environments, with QE values of − 81.5, − 46.6 and − 190.7 in E1, E2 and E4, respectively. This suggests that expression of QBGL-3 has similar effect under all tested environments. The QE interaction effects of these QTL were substantially smaller than the corresponding main effects (Table 4). This suggests that the genetic effects of these loci were largely controlled by their main effect and thus they could be considered as primary targets in future breeding programs. In addition, QE effects were observed for 18 QTL with no significant main effect. The contribution of phenotype variations explained by QE interactions for these QTL ranged from 0.2 to 9.1%. The highest number of QE interactions (8 loci) was detected for the SNI, suggesting that this trait was highly sensitive to environment. The QSNI-3-2 with the largest effect, accounting for 9.1% of the phenotypic variance, demonstrated changes in magnitude of effect; while QSNI-5-2 had differential expression between E1 and E2, demonstrating negative and positive QE effects, respectively.

### Candidate genes within the 3H QTL cluster

A number of coincidental QTL affecting two seed quality traits and five malt characters were identified on the short arm of chromosome 3H. These loci explained a relatively large proportion of phenotypic variation, in particular QSNI-3-1, QFRI-3 and QBGL-3 accounted for 27, 34 and 37% of phenotypic variance, respectively. Defined QTL cluster occupied relatively small chromosomal region and comprised at once five loci controlling variation of important malting quality characteristics. That makes it valuable for using in future breeding programs through marker assisted selection. In order to reveal possible candidate genes underlying identified QTL, the marker BK_08 associated with majority of detected loci was located on the POPSEQ genetic map of Morex × Barke [40]. The morex_contig_1580005, bearing the sequence of BK_08, was mapped at position 45.4 cM. The list of all annotated genes in the region between 40.4–50.4 cM was downloaded from publicly available BARLEX database [41]. Even a rough annotation-based assay of the genes located in this region (Additional file 5: Table S3) revealed a number of well-known malting quality related genes. These are the genes involved in starch, protein, lipid and cell wall metabolism. For instance, two genes MLOC_68194.1 and MLOC_36529.1 encoding aspartyl and serine proteases were found to be present in selected region. These two proteinase types are known to be involved in storage protein solubilisation, as well as in the regulation of the starch degrading enzyme, ß-amylase [42, 43]. In addition, four genes (AK375791, MLOC_32229.1, AK355146, AK375970) coding for heat shock proteins (HSP) were present in the region of interest. HSP might play a crucial role in protection of enzymes from degradation during the malting process especially at the stage of kilning. Association of HSP genes with such malting quality traits as malt extract, ß-amylase activity, friability, free amino acids and Kolbach Index have been previously reported [44, 45]. Interestingly, the target region also contained the gene MLOC_6671.1 encoding serine carboxypeptidase (Ser-CP). Ser-CPs play an important role in hydrolysis of storage proteins in germinating barley seeds. At least 6 members in barley were previously identified, all of which were found to be active during germination [46]. Transcript profiling of the gene Cxp1 (MLOC_55542.1), mapped to long arm of chromosomes 3H, in the Steptoe/Morex population revealed that expression QTL for respective gene co-localized with QTL affecting diastatic power [47]. Moreover, the region contains 18 genes encoding different types of transcription factors (TFs). They also could be considered as probable candidate genes, as these TFs might control expression of structural or regulatory genes playing key role in malting process. The fact that the members of the MYB gene family were found to be present in the list of potential candidates is particularly interesting. It has been previously reported that a MYB protein (GAMYB) expressed in cereal aleurone cells triggered transcriptional activation of α-amylase gene through binding to the TAACAAA box in its promoter [48]. The presence of elements of gibberellin responsive complex (GARC) have been reported within promoter regions of some other hydrolytic enzymes such as (1–3, 1–4)-β-glucanase and cathepsin B-like protease [49, 50]. Moreover, Gubler et al. [51] in a series of transient expression experiments showed that GAMYB protein indeed was able to promote expression of reporter gene fused with promoters of genes encoding EII(1–3, 1–4)-β-glucanase and cathepsin B-like protease.

## Conclusions

In this study we have investigated agronomic, malting and seed quality characteristics of two high yielding elite breeding lines that differ in response to drought stress. Our QTL mapping revealed several novel loci controlling malting quality which could be exploited in breeding programs. In this context, the complex QTL region on chromosome 3H seems of particular interest, as it harbors several large effect QTL, which need to be validated in different genetic backgrounds. Moreover, identification and validation of the candidate genes will help to select for intralocus recombinants, since recombination of favorable alleles is required for further optimization of the genetic make-up of this locus.

## Methods

### Plant materials and field experiments

Our study is based on a population of 100 unique DH lines developed from the cross between the elite spring barley lines Sofiara and Victoriana (KWS, Germany). The parental lines were selected because they largely differ with respect to YLD, TGW, SNI and, BGL, and their response to drought stress. The DH lines and their parents were evaluated in replicated field trials at three locations in 2 years (Table 6; Additional file 6: Figure S3). The experimental design followed a lattice (Wohlde, Walewice) or a randomized complete block design (Gatersleben). The plot sizes ranged from 0.4 m^2^ to 5.5 m^2^. In Gatersleben, terminal drought stress was applied 1 week after anthesis using a rain-out shelter in addition to a control variant with absence of drought stress. Soil moisture was monitored in the drought stress trials twice a week in 10, 20, 30 and 40 cm depths by using 82 evenly distributed soil moisture sensors (SM300, PR2/4; Delta T devices Ltd., England).

**Table 6:** Environments in which the Sofiara/Victoriana DH population was evaluated

Code	Number of plot replications	Environments	Plot size
E1	3	WW, Gatersleben, Germany, April–August, 2012	0.4 m^2^
E2	4	DS, Gatersleben, Germany, April–August, 2012	0.4 m^2^
E3	2	RF, Wohlde, Germany, April–August, 2011	5.0 m^2^
E4	2	RF, Wohlde, Germany, April–August, 2012	5.0 m^2^
E5	2	RF, Walewice, Poland, April–August, 2011	5.0 m^2^
E6	2	RF, Walewice, Poland, April–August, 2012	5.5 m^2^

WW, DS, and RF well watered, drought stress and rainfed conditions, respectively

### Yield-related traits

Plant height was measured before harvest from the base to the top of the plant and recorded in centimeters. SL, SB, SA and TGW were assessed using a sample of 400–500 grains and a digital seed analyser (Marvin; GTA Sensorik GmbH, Germany).

### Starch, carbon and nitrogen content

Soluble carbohydrates were extracted three times with 80% ethanol at 60 °C from 0.4 g of homogenized grains tissue and ethanol-insoluble pellet was used for the quantification of starch content. Pellet was solubilized in 1 N KOH for 1 h at 95 °C. After neutralization with 5 N HCl, starch hydrolysis was achieved by incubation with amyloglucosidase (6u/ml) and a-amylase (10 u/ml) at 37 °C for 90 min. Glucose was quantified using enzymatic assay kit (R-Biopharm AG, Darmstadt, Germany). Carbon and nitrogen content were determined in ground and oven dried samples of grains using an elemental analyzer (vario EL III; Elementar analysensysteme GmbH Hanau, Germany).

### Malting quality analysis

Grain samples (80 g per genotype) were malted in the malting facilities of the Research Institute for Raw Materials at the VLB Berlin e.V.. Micromalting conditions were the following: 5 h wet steep, 19 h air-rest, 2 h wet steep, 22 h air-rest, spray steeping until reaching a final moisture content of 45%, then germination for 96 h at 14.5 °C and 95 to 98% relative air humidity. Germinated samples were kilned with the following regime: 16 h at 50 °C, 1 h at 60 °C, 1 h at 70 °C and 5 h at 80 °C.

To assess malt quality following twelve parameters such as malt moisture content (MMC), FGE, VIS, PRO, SNI, KOL, FLA, FRI, BGL, TML, REL and RTL were determined according to the methods of MEBAK (Mitteleuropäische Brautechnische Analysenkommission). A brief outline of some malting traits is given in Potokina et al. [44].

### Genotyping and construction of the genetic map

DNA samples from leaf tissue of the DH lines were extracted using the DNeasy Plant DNA miniprep kit (Qiagen, Germany), and scored with barley 9 K SNP iSelect Illumina array. Genetic map was constructed using the software package JoinMap 4.0 [52]. Linkage groups were established using minimum LOD values of 4. Monte Carlo maximum likelihood (ML) mapping algorithm was applied to determine the orders of markers within each linkage group. Recombination frequencies were converted to centimorgans (cM) using Haldane’s mapping function. The order of markers was compared with a published map [40].

### Phenotypic data analysis and QTL mapping

We performed a two-stage analysis of phenotypic data [53]. The BLUEs of all genotypes in each environment were combined and a linear mixed model across environments was fitted with random genotype, environment, and residual effects. All linear mixed models were implemented using ASReml-R [54]. For QTL mapping we applied a composite mapping (CM) approach [55, 56]. For more details see Additional file 7: Data S1.

## Additional files

Additional file 1: **Figure S1.** Frequency distribution of the BLUEs of the 100 DH for the measured traits. (PDF 257 kb)

Additional file 2: **Table S1.** Genetic positions of markers. (XLSX 48 kb)

Additional file 3: **Table S2.** The collective percent of phenotypic variation explained by main-effect QTL and QE interactions for the trait. (XLSX 12 kb)

Additional file 4: **Figure S2.** Locations of main-effect QTL on 3H (a) and 4H (b) chromosomes. (PDF 183 kb)

Additional file 5: **Table S3.** The list of candidate genes associated with largest QTL cluster on chromosome 3H. (XLSX 34 kb)

Additional file 6: **Figure S3.** Climate conditions in rainfed field located in Wohlde (a-b), Walewice (c-d) and Gatersleben (e). (PDF 409 kb)

Additional file 7: **Data S1.** Experimental procedures related to phenotypic data analysis, and QTL mapping [57–61]. (DOCX 14 kb)

## Abbreviations

BGL: β-glucan
FGE: Fine grind extract
FLA: Limit of attenuation
FRI: Friability
FT: Flowering time
KOL: Kolbach index
LOD: Logarithm of odds
MAS: Marker-assisted selection
MMC: Moisture content
PH: Plant height
PRO: Protein content
QTL: Quantitative trait locus
REL: Respiration losses
RTL: Rootlet losses
SA: Seed area
SB: Seed breadth
SCC: Seed carbon content
SL: Seed length
SNC: Seed nitrogen content
SNI: Soluble nitrogen
SNP: Single nucleotide polymorphism
SSC: Seed starch content
TGW: Thousand-grain weight
TML: Total malting losses
VIS: Viscosity
YLD: Grain yield
