# Supplementary material for: Identification of QTL hot spots for malting quality in two elite breeding lines with distinct tolerance to abiotic stress
Source: BMC Plant Biol. 2018 Jun 4;18:106. doi: 10.1186/s12870-018-1323-4 (PMC5987402; doi:10.1186/s12870-018-1323-4)
Supplement: Supplementary file 1 — Figure S1. Frequency distribution of the BLUEs of the 100 DH for the measured traits. (PDF 257 kb) [file 12870_2018_1323_MOESM1_ESM.pdf]

Additional file 1: Figure S1. Frequency distribution of the BLUEs of the 100 DH for the measured traits. The black arrows indicate the values of parents Sofiara (S) and Victoriana (V).

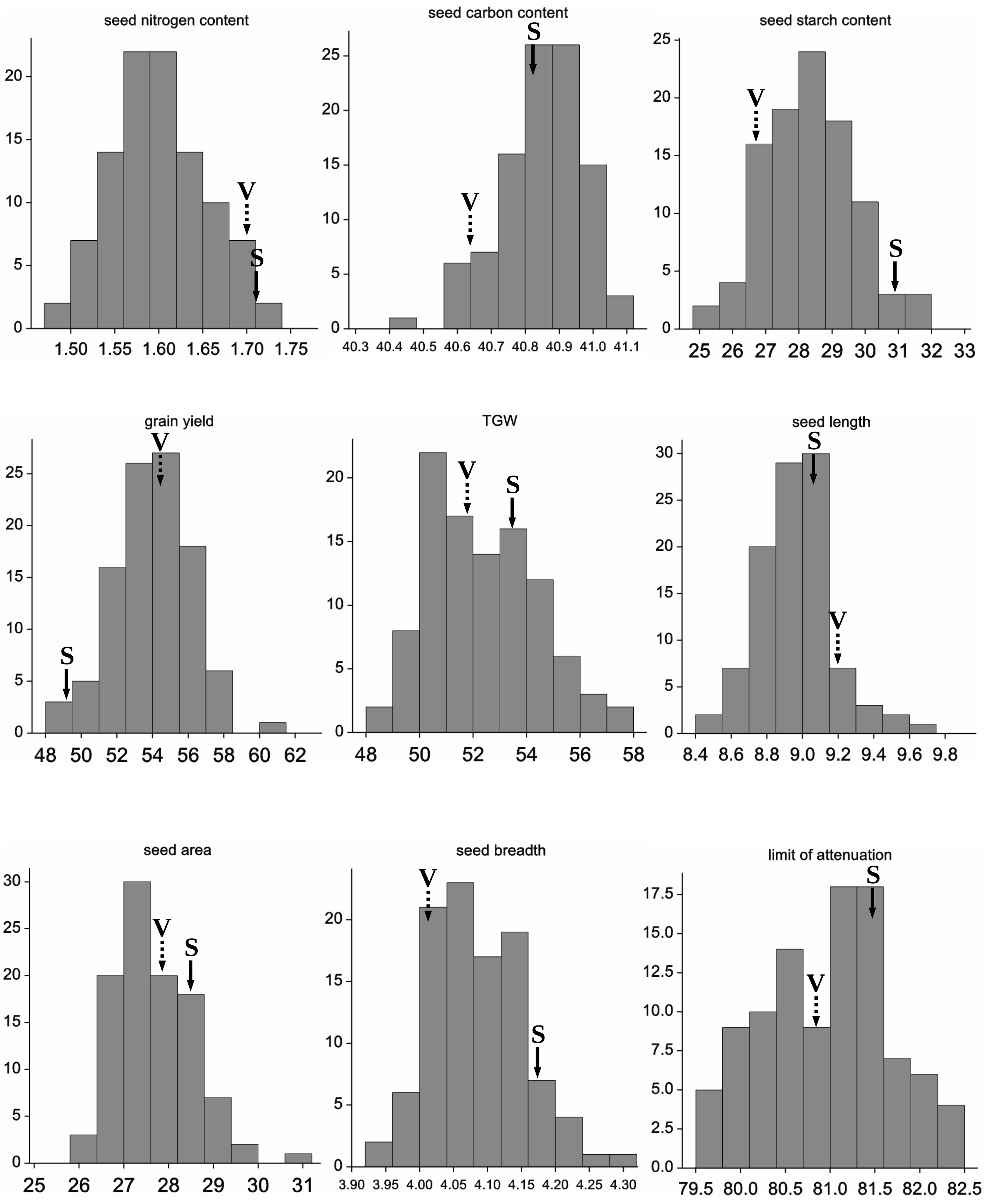

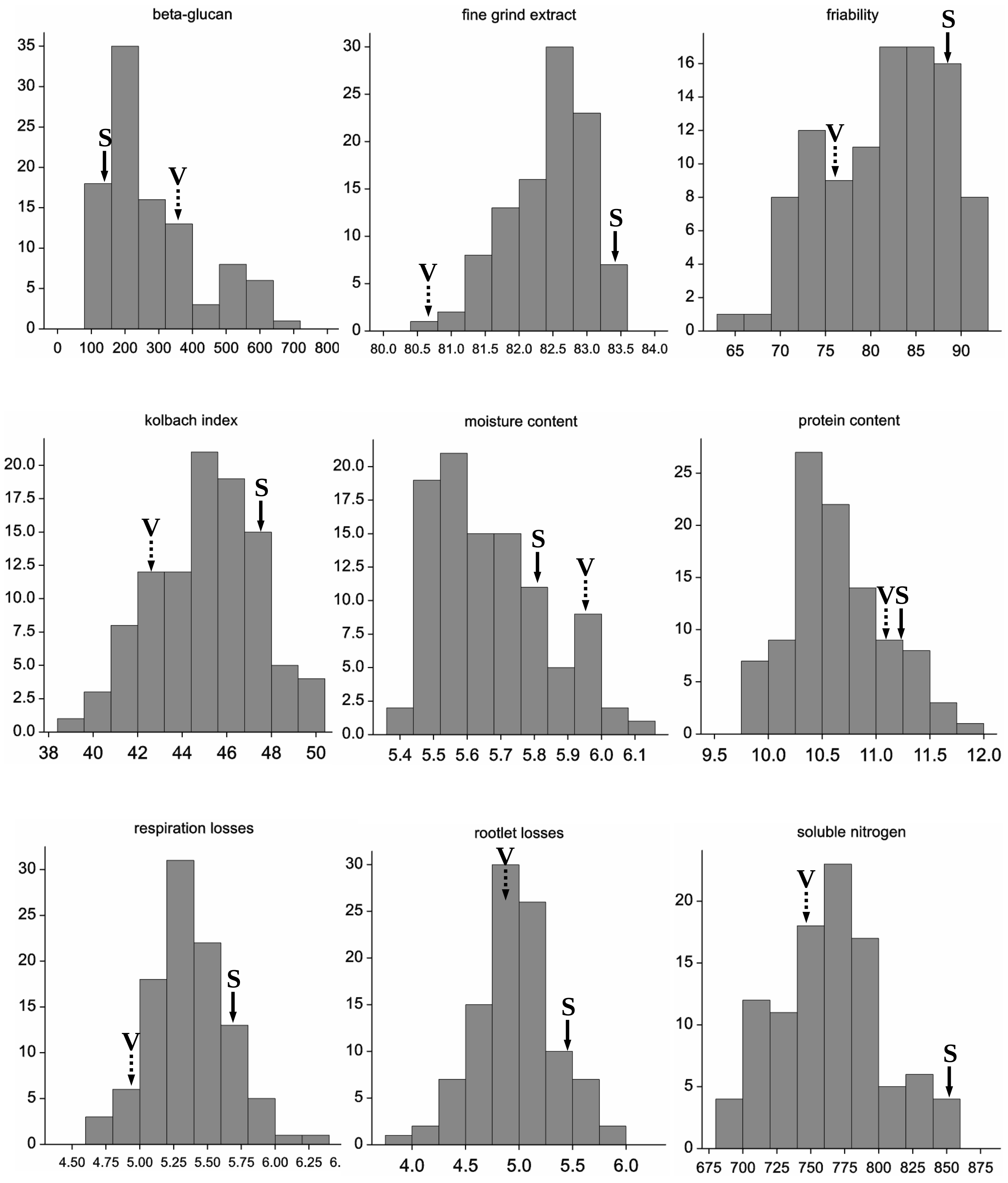

beta-glucan
fine grind extract
friability
kolbach index
moisture content
protein content
respiration losses
rootlet losses
soluble nitrogen
S
V

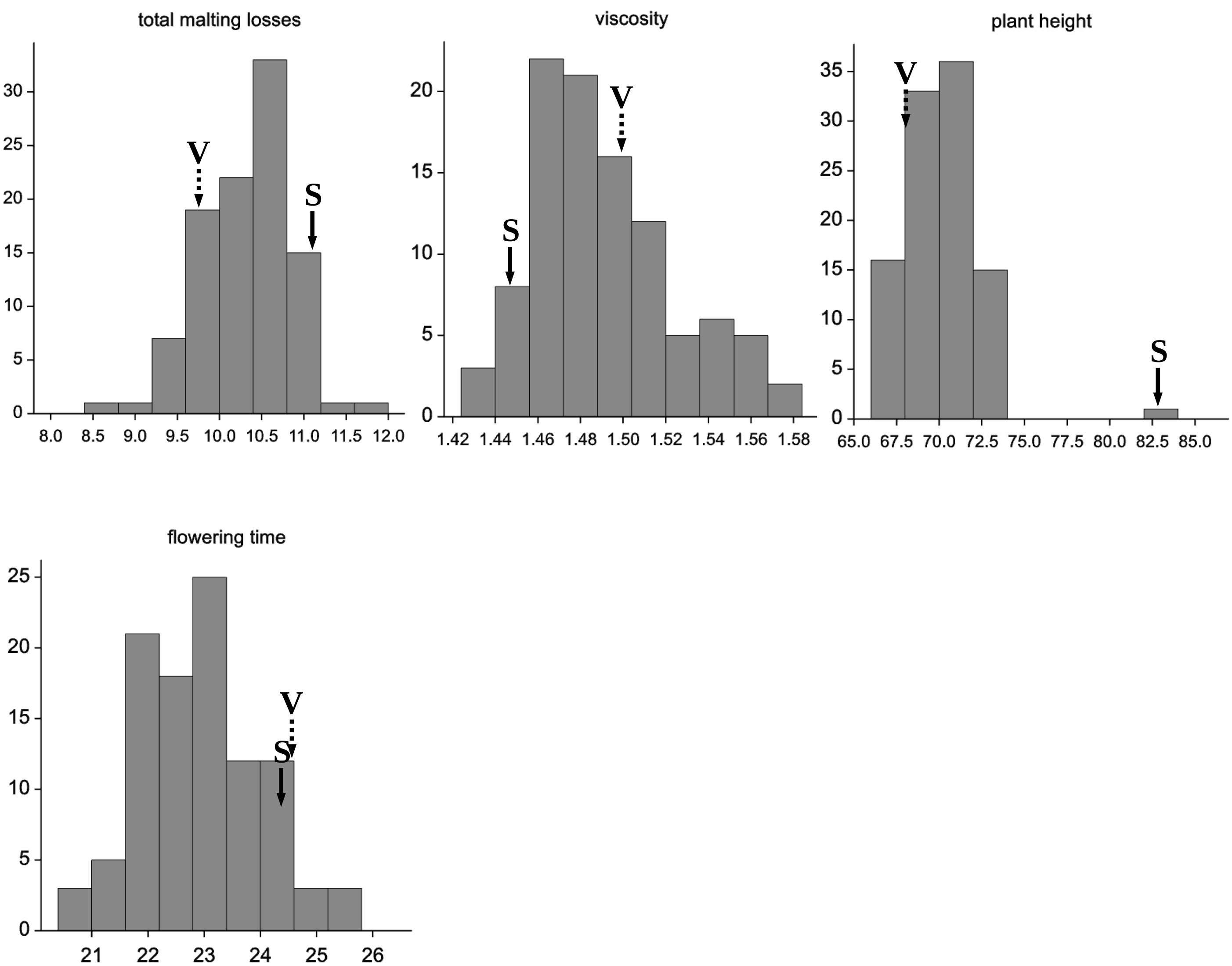
total malting losses
V
S
0
5
10
15
20
25
30
8.0
8.5
9.0
9.5
10.0
10.5
11.0
11.5
12.0
viscosity
V
S
0
5
10
15
20
1.42
1.44
1.46
1.48
1.50
1.52
1.54
1.56
1.58
plant height
V
S
0
5
10
15
20
25
30
35
65.0
67.5
70.0
72.5
75.0
77.5
80.0
82.5
85.0
flowering time
V
S
0
5
10
15
20
25
21
22
23
24
25
26
