# Supplementary figures and images for: Identification of QTL hot spots for malting quality in two elite breeding lines with distinct tolerance to abiotic stress
Source: BMC Plant Biol. 2018 Jun 4;18:106. doi: 10.1186/s12870-018-1323-4 (PMC5987402; doi:10.1186/s12870-018-1323-4)

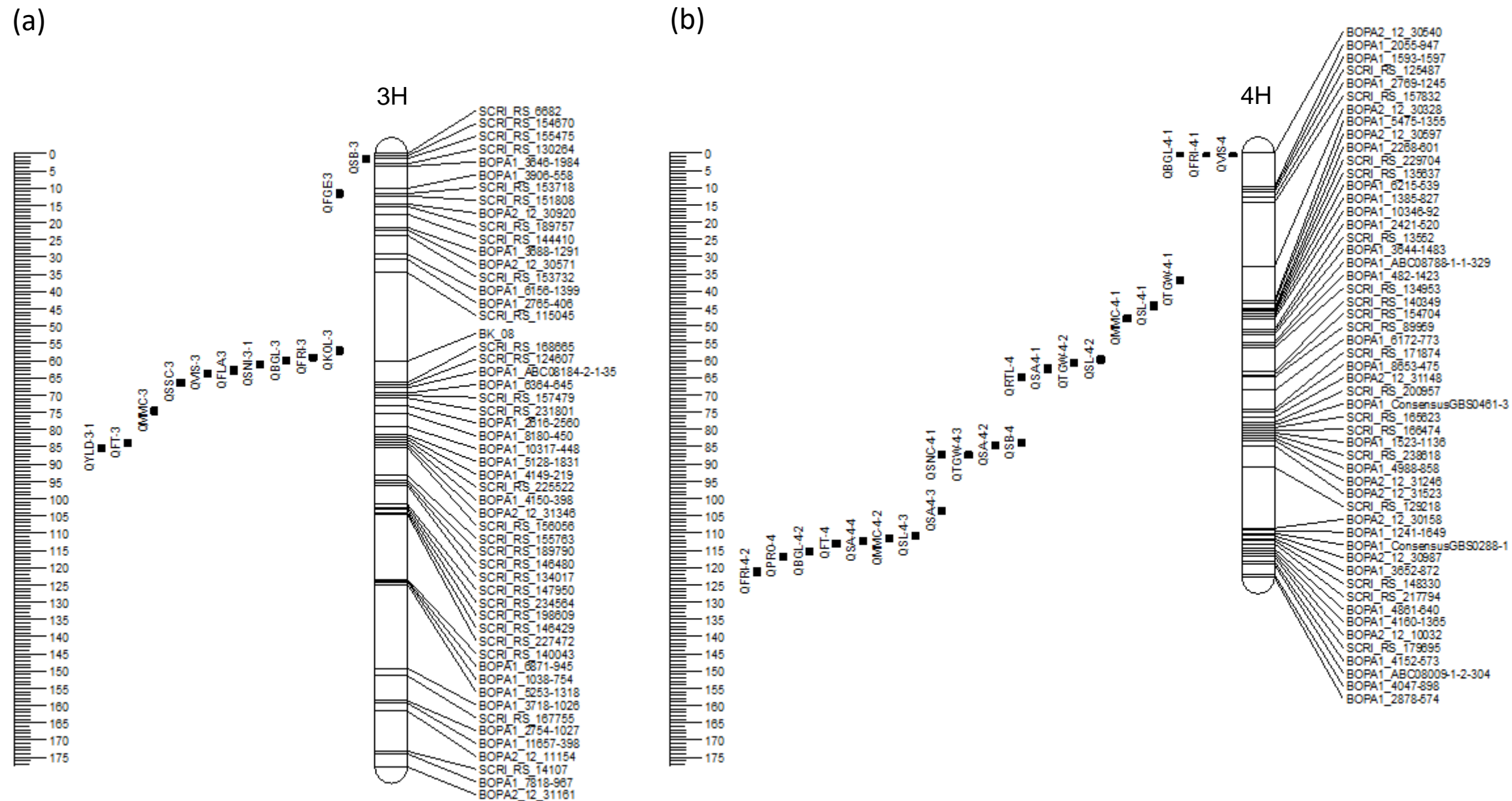

Additional file 2: Figure S2. Locations of main-effect QTL on 3H (a) and 4H (b) chromosomes.

Supplement: Supplementary file 4 — Figure S2. Locations of main-effect QTL on 3H (a) and 4H (b) chromosomes. (PDF 183 kb) [file 12870_2018_1323_MOESM4_ESM.pdf]
