# Supplementary material for: Identification of QTL hot spots for malting quality in two elite breeding lines with distinct tolerance to abiotic stress
Source: BMC Plant Biol. 2018 Jun 4;18:106. doi: 10.1186/s12870-018-1323-4 (PMC5987402; doi:10.1186/s12870-018-1323-4)
Supplement: Supplementary file 6 — Figure S3. Climate conditions in rainfed field located in Wohlde (a-b), Walewice (c-d) and Gatersleben (e). (PDF 409 kb) [file 12870_2018_1323_MOESM6_ESM.pdf]

Additional file 3: Figure S3. Climate conditions in rainfed field located in Wohlde (a-b), Walewice (c-d) and Gatersleben (e). Moisture content of soil in the field in Gatersleben location (f). Bars represent rainfall and lines – Temperature (a-e).

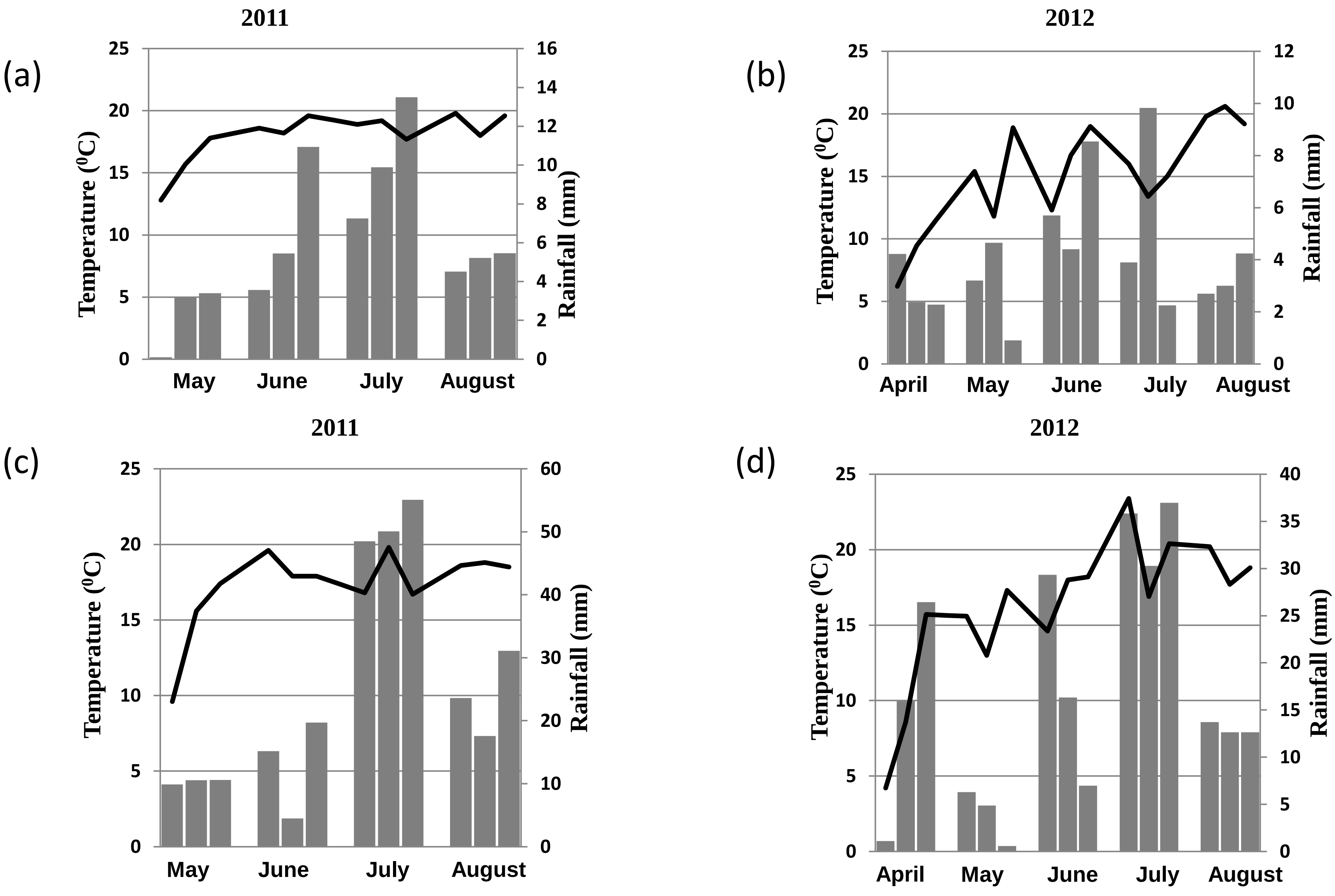

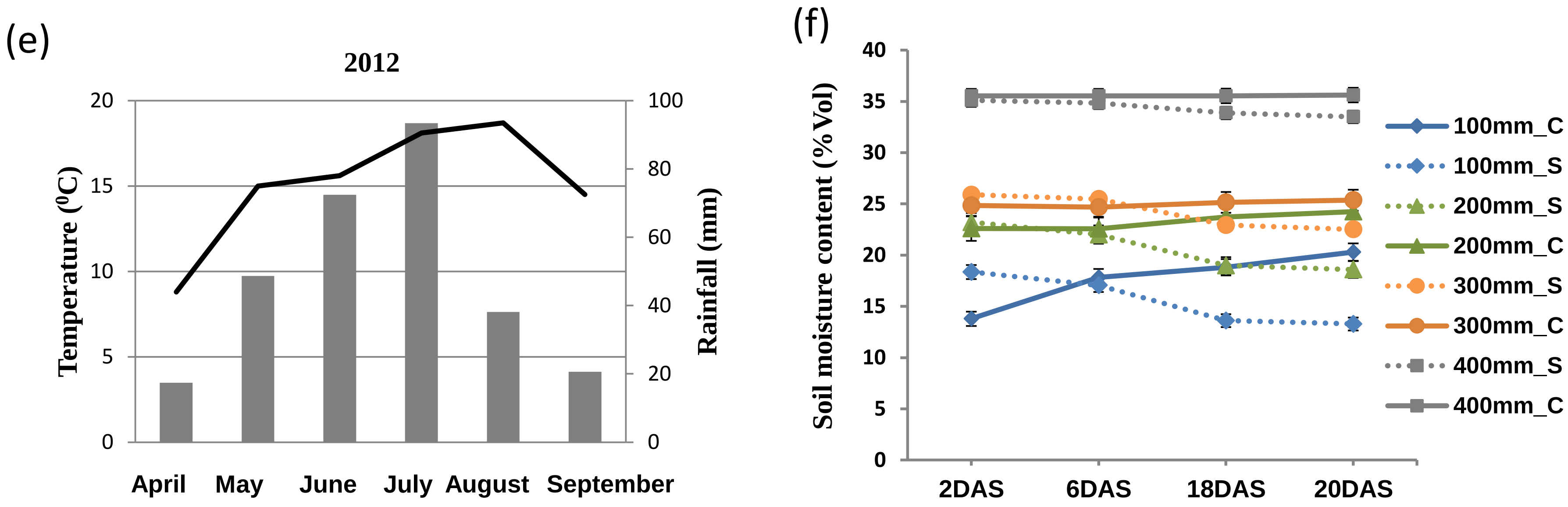
(e)
2012
Temperature (0C)
Rainfall (mm)
0
5
10
15
20
0
20
40
60
80
100
April
May
June
July
August
September
(f)
Soil moisture content (% Vol)
0
5
10
15
20
25
30
35
40
2DAS
6DAS
18DAS
20DAS
100mm_C
100mm_S
200mm_S
200mm_C
300mm_S
300mm_C
400mm_S
400mm_C
