# Supplementary material for: Identification of QTL hot spots for malting quality in two elite breeding lines with distinct tolerance to abiotic stress
Source: BMC Plant Biol. 2018 Jun 4;18:106. doi: 10.1186/s12870-018-1323-4 (PMC5987402; doi:10.1186/s12870-018-1323-4)
Supplement: Supplementary file 7 — Data S1. Experimental procedures related to phenotypic data analysis, and QTL mapping [57–61]. (DOCX 14 kb) [file 12870_2018_1323_MOESM7_ESM.docx]

**Additional file 7: Data S1.** Experimental procedures related to phenotypic data analysis, and QTL mapping.

**Phenotypic data analysis**

We performed a two-stage analysis of phenotypic data (Möhring and Piepho, 2009). At the first stage, we analyzed the data for each environment separately using a linear mixed model assuming genotype, replication, incomplete block and residual as random effects. For the environments where a complete randomized block design was used, incomplete block effect was not considered. Variance components were estimated and the repeatability for each environment was calculated as: $\frac{\sigma_{G}^{2}}{\sigma_{G}^{2}+\frac{\sigma_{e}^{2}}{R}}$, where $\sigma_{G}^{2}$ is the genotypic variance, $\sigma_{e}^{2}$ is the residual variance and $R$ is the average number of replications per genotype. To obtain the best unbiased linear estimation (BLUE) for each genotype, we fitted another linear mixed model with fixed genotypic effects and all other effects remain random.

At the second stage, the BLUEs of all genotypes in each environment were combined and a linear mixed model across environments was fitted with random genotype, environment, and residual effects. Variance components were used to estimate broad-sense heritability as $h^{2}=\frac{\sigma_{G}^{2}}{\sigma_{G}^{2}+\frac{\tilde{\sigma}_{e}^{2}}{E}}$, where $\sigma_{G}^{2}$ is the genotypic variance, $\tilde{\sigma}_{e}^{2}$ is the residual variance, and $E$ refers to the average number of environments where a genotype has been tested. In addition, genotypic effects were assumed as fixed to obtain the BLUEs for each genotype across environments. All linear mixed models were implemented using ASReml-R (Gilmour et al. 2009).

**QTL mapping considering QTL-by-environment interaction**

For QTL mapping we applied a composite mapping (CM) approach (Jansen 1994, Zeng 1994). In particular, QTL-by-environment interaction was taken into account following Jansen et al. (1995). Cofactors were selected using a step-wise multiple linear regression model. We started from an initial model in which the dependent variable was the phenotypic record (BLUEs) of each genotype in each environment and the independent variables consisted of an intercept term and environment effects. Then the effects of all markers as well as all possible marker-by-environment interaction effects entered the model one-by-one and variables were selected with the Bayesian information criterion (BIC, Schwarz 1978). The set of cofactors consisted of the variables remaining in the final model.

To detect main effect QTL, we compared the maximum likelihood $L_{1}$ of the model containing all cofactors as well as the main effect of the putative QTL and the maximum likelihood $L_{0}$ of the model containing cofactors only. Then a likelihood ratio test was performed based on the test statistic $LR=2\log_{e}(L_{1}/L_{0})$, which asymptotically follows a $\chi^{2}$-distribution. Note that an equivalent test can be performed based on the logarithm of odds (LOD) score $\log_{10}(L_{1}/L_{0})$. To detect significant QTL-by-environment interaction, another likelihood ratio test was applied based on $LR'=2\log_{e}(L_{2}/L_{1})$, where $L_{2}$ denoted the maximum likelihood of the model containing cofactors as well as the main and environment-interaction effect of the putative QTL.

A genome-wide threshold for p-values or LOD scores was obtained by performing a permutation test (Churchill and Doerge 1994) for each trait. Peak positions of putative QTL and QTL-by-environment interactions that passed the threshold were recorded. A multiple linear regression model was re-fitted with all significant QTL and QTL-by-environment interactions to estimate their R-squares and main effects of each QTL, as well as QTL-by-environment interaction effects.

Finally, we estimated the cross-validated total R-square contributed by all significant QTL and QTL-by-environment interactions for each trait. We applied a five-fold cross-validation procedure with a total of 100 different combinations of estimation and test sets. In each fold of cross-validation, the genotypes were randomly divided into five subsets. Four of the five subsets were used as the estimation set and the remaining one formed the test set. QTL mapping was performed in the estimation set and the effects of significant QTL and QTL-by-environment interaction were estimated, which were then used to predict the performance of the genotypes in the test set. The cross-validated R-square was calculated as the squared Pearson product-moment correlation between predicted and observed values of the genotypes in the test set. After 100 runs of cross-validation, the averaged R-square was finally recorded as the total R-square contributed by all significant QTL and QTL-by-environment interactions.

Selecting of cofactors was implemented using PROC MIXED in SAS software (SAS Institute 2009). All remaining programs for QTL mapping were implemented using the R software (R core team 2014).
